# Supplementary material for: Clinical features and outcomes of hospitalised patients with COVID-19 and Parkinsonian disorders: A multicentre UK-based study
Source: PLoS One. 2023 Jul 31;18(7):e0285349. doi: 10.1371/journal.pone.0285349 (PMC10389727; doi:10.1371/journal.pone.0285349)
Supplement: S1 File — (PDF) [file pone.0285349.s001.pdf]

Using this document.

A picture in a blue box details the data entry form seen in the survey. There may be explanations or notes. Where relevant these are highlighted on the form. (Please see the entry page to the study below showing this.)

Throughout the survey there will be questions; that when answered with certain selections, will open secondary questions expecting a response. Please check that all questions have been completed where appropriate and possible. Where a selection list can be used in the survey an example is included in this document.

Required data has been highlighted on the survey page and the survey will not allow data entry to continue until this question has been answered. If there are any sections where you come across a problem or have a required question but cannot answer please contact the study team.

At the top of the survey you will see a bar that indicates your progress and the percentage of the survey that has been completed.

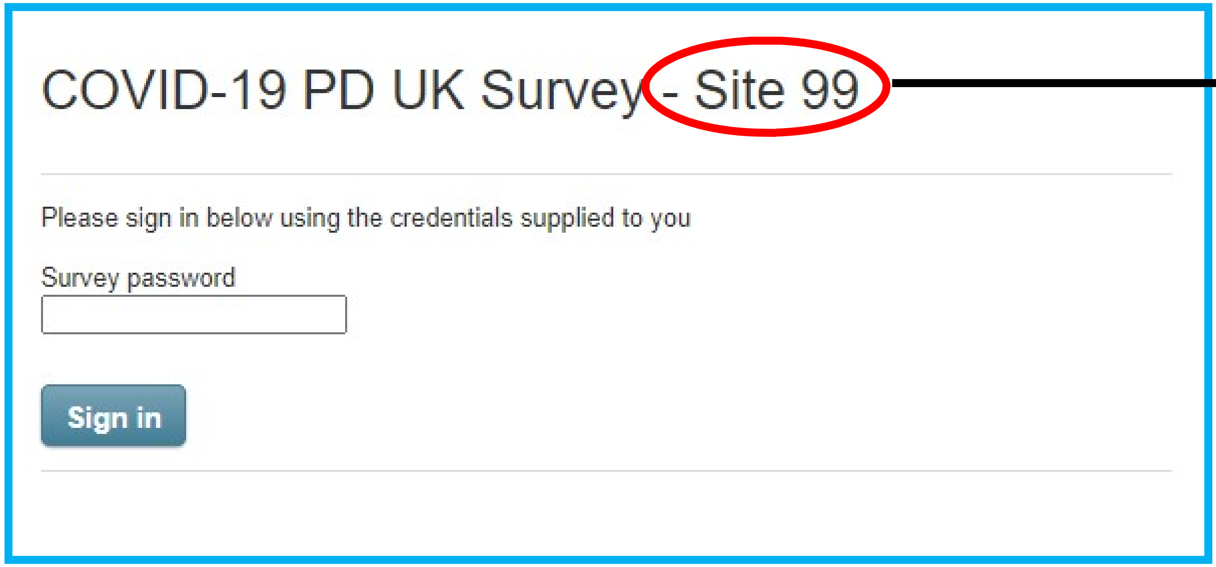

Please note: this is an example name.

You will see a two number code representing your site here instead of “Site 99”.

Please check that you have the correct code according to the registration email for your site.

Please sign in with the password supplied to the person registering the site.

Each site has a unique identifier and password.

Introduction and Consent Statement

In this survey we would like to capture information about people with Parkinson's syndromes who have tested positive with COVID-19 in the hospital setting.

Please provide an identifier for the participant that is kept at site and do not include any personal details.

We are capturing details about patients who have tested positive to COVID-19. There are 4 sections:

1. Patient information
2. Admission information
3. Course of COVID-19
4. Outcome

Consent Statement

I confirm that I have read the information sheet dated 17th November 2020 (version 2.0) and any questions I have raised have been answered to my satisfaction.

I understand that my participation is voluntary and I can choose to not take part at any time by not continuing further with this survey.

I understand that the data collected is solely for research purposes.

I agree to take part in this study. ✱ *Required*

○ I agree

To enter a patient, please click Next.

If you encounter any problems or have any questions please contact jemma.inches@plymouth.ac.uk

Next ›

Please note: The next button will appear at the end of each page.

If the next page does not load—please check that you have answered each required question.

If a required question has been missed the colour will change from this: ✱ *Required* to: **! Required**

Patient Demographics

Patient Identifier (*to be allocated by site and stored locally - please do not use any personal identifying information*). **Please refer to your registration email for guidance on patient indentifier creation.** ✱ *Required*

Your answer should be no more than 10 characters long.

Year of birth ✱ *Required*

Sex ✱ *Required*

- ○ Female
- ○ Male

Ethnicity ✱ *Required*

Please select

- White - English / Welsh / Scottish / Northern Irish / British
- White - Irish
- White - Gypsy or Irish Traveller
- White - Any other White background
- Mixed Race - White and Black Caribbean
- Mixed Race - White and Black African
- Mixed Race - White and Asian
- Mixed Race - Any other mixed / Multiple Ethnic background
- Asian / Asian British - Indian
- Asian / Asian British - Pakistani
- Asian / Asian British - Bangladeshi
- Asian / Asian British - Any other Asian background
- Chinese
- Black or Black British - African
- Black or Black British - Caribbean
- Black or Black British - Any other Black background
- Arab
- Any other ethnic group

Location pre-admission ✱ *Required*

- ○ Own Home/private residence
- ○ Residential or nursing home
- ○ Local/community hospital
- ○ Other

If you selected Other, please specify: **Required**

Your answer should be no more than 35 characters long.

Please Note: This page is continued overleaf

Please note:

From this page a ‹ Previous button will appear in the lower left corner allowing the return to the preceding page. The information that has been added will be saved if this back button is clicked.

Please Note: This page is a continuation of the previous form

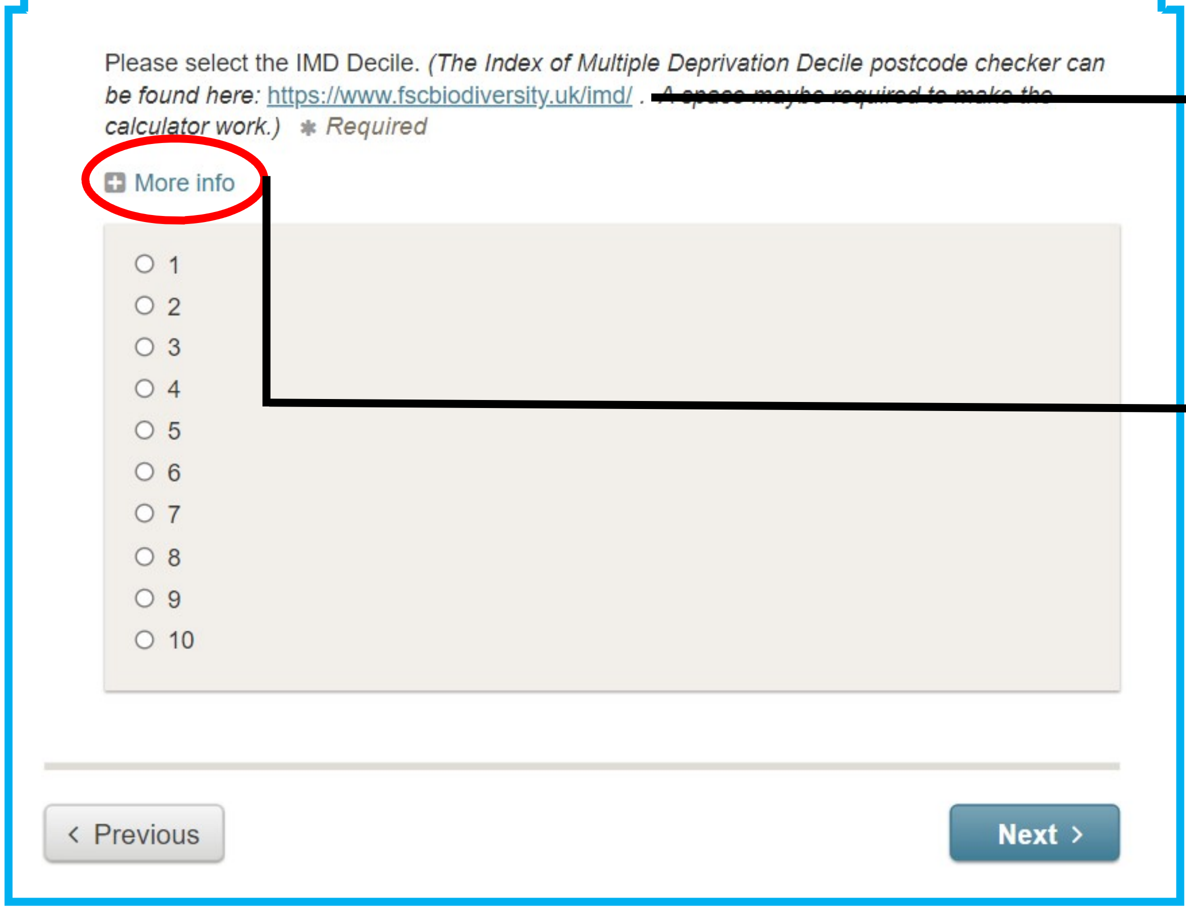

Please select the IMD Decile. *(The Index of Multiple Deprivation Decile postcode checker can be found here:* https://www.fscbiodiversity.uk/imd/ . *A space maybe required to make the calculator work.)* * *Required*

More info

- 1
- 2
- 3
- 4
- 5
- 6
- 7
- 8
- 9
- 10

‹ Previous | Next ›

Please note: The hyperlink opens a new web browser page to the Index of Multiple Deprivation (IMD) Decile calculator.

Please check for spaces between the first and last part of the post-code or the calculator will not work correctly.

Please note: The more info button expands to show the following information about the IMD

The Index of Multiple Deprivation, commonly known as the IMD, is the official measure of relative deprivation for small areas in England.

The Index of Multiple Deprivation ranks every small area, called lower-layer super output areas (LSOA), in England from 1 (most deprived area) to 32,844 (least deprived area).

The IMD combines information from the seven domains to produce an overall relative measure of deprivation. The domains are combined using the following weights:

- Income Deprivation (22.5%)
- Employment Deprivation (22.5%)
- Education, Skills and Training Deprivation (13.5%)
- Health Deprivation and Disability (13.5%)
- Crime (9.3%)
- Barriers to Housing and Services (9.3%)
- Living Environment Deprivation (9.3%)

Parkinson's Syndrome Information

Year of first diagnosis of Parkinson's syndrome ✱ *Required*

Please note: This date relates to the first Parkinson's syndrome that someone may have.

Diagnosis on Admission ✱ *Required*

- ○ Parkinson's disease
- ○ Parkinson's disease dementia
- ○ Dementia with Lewy bodies
- ○ Multiple system atrophy
- ○ Progressive supranuclear palsy
- ○ Corticobasal degeneration
- ○ Other

This part of the survey uses a table of questions, view as separate questions instead?

Is the individual known to have any of the following features associated with their Parkinson's syndrome (**prior to this current illness episode**)?

	✱ *Required*		
	yes	no	unknown
Significant cognitive impairment (dementia/psychosis impacting on quality of life with or without treatment)	○	○	○
Bulbar symptoms sufficient to cause swallowing difficulty (beyond drooling/hypophonia)	○	○	○
Significant respiratory compromise (requiring home oxygen, CPAP, NIV or suction)	○	○	○
Evidence of significant autonomic neuropathy (symptomatic or significant orthostatic hypotension despite treatment)	○	○	○
Marked motor fluctuations with severe disability/frailty in the OFF state	○	○	○

Please Note: This page is continued overleaf

Please Note: This page is a continuation of the previous form

Clinical Frailty Score * *Required*

Less info

Clinical Frailty Score

- <5
- 5
- 6
- 7
- 8
- 9

Hoehn and Yahr stage (**prior to this illness episode**)

Please select

< Previous

Next >

Please note: The more info button expands to show a link that opens a new web browser page to the clinical frailty scale as shown below.

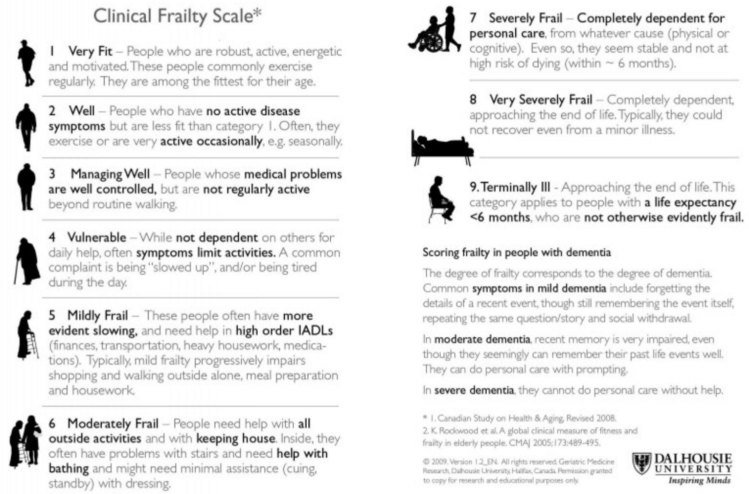

Clinical Frailty Scale*

1 **Very Fit** – People who are robust, active, energetic and motivated. These people commonly exercise regularly. They are among the fittest for their age.

2 **Well** – People who have **no active disease symptoms** but are less fit than category 1. Often, they exercise or are very **active occasionally**, e.g. seasonally.

3 **Managing Well** – People whose **medical problems are well controlled,** but are **not regularly active** beyond routine walking.

4 **Vulnerable** – While **not dependent** on others for daily help, often **symptoms limit activities.** A common complaint is being "slowed up", and/or being tired during the day.

5 **Mildly Frail** – These people often have **more evident slowing,** and need help in **high order IADLs** (finances, transportation, heavy housework, medications). Typically, mild frailty progressively impairs shopping and walking outside alone, meal preparation and housework.

6 **Moderately Frail** – People need help with **all outside activities** and with **keeping house**. Inside, they often have problems with stairs and need **help with bathing** and might need minimal assistance (cuing, standby) with dressing.

7 **Severely Frail** – **Completely dependent for personal care**, from whatever cause (physical or cognitive). Even so, they seem stable and not at high risk of dying (within ~ 6 months).

8 **Very Severely Frail** – Completely dependent, approaching the end of life. Typically, they could not recover even from a minor illness.

9. **Terminally Ill** - Approaching the end of life. This category applies to people with **a life expectancy <6 months**, who are **not otherwise evidently frail.**

Scoring frailty in people with dementia

The degree of frailty corresponds to the degree of dementia. Common **symptoms in mild dementia** include forgetting the details of a recent event, though still remembering the event itself, repeating the same question/story and social withdrawal.

In **moderate dementia**, recent memory is very impaired, even though they seemingly can remember their past life events well. They can do personal care with prompting.

In **severe dementia**, they cannot do personal care without help.

* 1. Canadian Study on Health & Aging, Revised 2008.
2. K. Rockwood et al. A global clinical measure of fitness and frailty in elderly people. CMAJ 2005;173:489-495.

© 2009. Version 1.2_EN. All rights reserved. Geriatric Medicine Research, Dalhousie University, Halifax, Canada. Permission granted to copy for research and educational purposes only.

DALHOUSIE UNIVERSITY *Inspiring Minds*

1. Unilateral involvement only
1.5 Unilateral and axial involvement
2. Bilateral involvement without impairment of balance
2.5 Mild bilateral disease with recovery on pull test
3 Mild to moderate bilateral disease; some postural instability; physically independent
4. Severe disability; still able to walk or stand unassisted
5. Wheelchair bound or bedridden unless aided
x. Information not available

Medications: Please list Parkinson's medications on admission

Please select all that apply * *Required*

- ☐ L-dopa (immediate, controlled, with entacapone and dispersible)
- ☐ COMT-I
- ☐ Amantadine
- ☐ Dopamine Agonist
- ☐ MAOB-I
- ☐ Anticholinergic
- ☐ Advanced Therapies
- ☐ Other Medication (not listed)
- ☐ Not Applicable

Which L-dopa preparations? **Required**

- ☐ Immediate release
- ☐ Controlled release
- ☐ With entacapone
- ☐ Dispersible

Immediate release total daily dose (levodopa component only). **Required**

Controlled release total daily dose (levodopa component only). **Required**

Please Note: This page is continued overleaf

Please note: As each medication type is selected further questions will follow underneath.

Please be aware that certain selections may have multiple options. These have been shown in the stepped boxes below.

Please Note: Apomorphine is under advanced therapies.

Please Note: Due to restricted programming of the survey, it is possible to select "Not Applicable" and other medication types.

As this is a required question, the "Not Applicable" has to be an available option, accidental selection will not delete medication data.

Please Note: Due to entacapone being given at the same time as an L-dopa medication (either as a pre-prepared medication or adjunctive medication—please include all entacapone doses under L-dopa ***not as COMT-I***.

Please Note: There is an appendix that lists common drug names and which group of medicines they come under: e.g. Madopar falls under L-Dopa. Please contact the COVID 19 PD UK study team if there are medications you are unsure of.

UNIVERSITY OF PLYMOUTH

Please Note: This page is a continuation of the previous form

Which L-dopa (with entacapone) preparations **Required**

Please select at least 1 answer(s).

- ☐ Immediate release
- ☐ Controlled release
- ☐ Dispersible

L-Dopa with entacapone (immediate release) total daily dose **Required**

Your answer should be no more than 5 characters long.

L-Dopa with entacapone (controlled release) total daily dose **Required**

Your answer should be no more than 5 characters long.

L-Dopa with entacapone (dispersible) total daily dose **Required**

Your answer should be no more than 5 characters long.

Dispersible total daily dose (levodopa component only). If dispersible l-dopa is only taken PRN, please enter minimum daily amount and acknowledge PRN with next question. **Required**

Is dispersible l-dopa PRN only? **Required**

- ○ Yes
- ○ No

Please Note: This page is continued overleaf

Please note: There may be validation rules for some elements. If you find that you have an inconsistency between source and data capture please contact the study team. An example of where incorrect data will flag on the survey is shown below

Example:

Amantadine total daily dose in mg

Please make sure the number is between 50 and 500.

Please Note: This page is a continuation of the previous form

Which COMT-I? *(Please note that entacapone should be placed under L-Dopa)* **Required**

- ○ Tolcapone
- ○ Opicapone

Tolcapone daily dose **Required**

- ○ 300mg
- ○ 600mg

Amantadine total daily dose in mg **Required**

[ ]

Dopamine agonists *(please note apomorphine can be found under advanced therapies)* **Required**

- ☐ Bromocriptine
- ☐ Cabergoline
- ☐ Pergolide
- ☐ Pramipexole
- ☐ Ropinirole
- ☐ Rotigotine
- ☐ Other

Total bromocriptine daily dose (mg)

Please enter a number.
Please make sure the number is between 1 and 30.
Your answer should be no more than 5 characters long.

[ ]

Please Note: This page is continued overleaf

Please note: There may be validation rules for some elements. If you find that you have an inconsistency between source and data capture please contact the study team. An example of where incorrect data will flag on the survey is shown below

Example:

Amantadine total daily dose in mg

Please make sure the number is between 50 and 500.

[ ]

Please Note: This page is a continuation of the previous form

Total cabergoline daily dose (mg)

Please enter a number.
Please make sure the number is between 0.5 and 3.
Your answer should be no more than 4 characters long.

Total pergolide daily dose in milligrams (please convert micrograms to milligrams)

Please enter a number.
Please make sure the number is between 0.05 and 3.
Your answer should be no more than 5 characters long.

Total pramiprexole daily dose (base in milligrams); please convert salt to base and micrograms to milligrams

Please enter a number.
Please make sure the number is between 0.088 and 3.15.
Your answer should be no more than 6 characters long.

Total ropinirole daily dose in mg (please convert micrograms to milligrams)

Please enter a number.
Please make sure the number is between 0.75 and 24.
Your answer should be no more than 4 characters long.

Please Note: This page is continued overleaf

Please note: Where possible validation information has been given to advise of data entry. If source data does not agree with data entry please contact the study team. Daily doses taken from the BNF.

Please Note: This page is a continuation of the previous form

Total rotigotine daily dose in mg

If other, please state name and total daily dose in mg

Your answer should be no more than 50 characters long.

Please note: This "if other, please state name and total daily dose" option is relevant only for dopamine agonists

Which MAOB-I? Required

- Rasagaline
- Selegeline
- Safinamide
- Other

Please note: Selecting Rasagaline will not open a further question for dose.

Please select selegeline total daily dose (mg)

Please select

1.25
5
10

Please select safinamide total daily dose Required

- 50mg
- 100mg

Which anticholinergic? Required

- Trihexyphenidyl (Benzhexol)
- Procyclidine
- Other

Please note: You cannot select more than one option

Please Note: This page is continued overleaf

Please Note: This page is a continuation of the previous form

Total trihexyphenidyl daily dose (mg)

Please enter a whole number (integer).
Please make sure the number is between 1 and 20.
Your answer should be no more than 2 characters long.

Total procyclidine daily dose (mg)

Please enter a number.
Please make sure the number is between 2.5 and 30.
Your answer should be no more than 3 characters long.

Which advanced therapies? Required

- ☐ DBS
- ☐ Duodopa
- ☐ Apomorphine
- ☐ Other

If other, please describe Required

If you selected Other Medication (not listed), please specify name and dose: Required

‹ Previous   Next ›

Please note: No dosages/settings are required

Please note: This "if you selected Other Medication (not listed)" option is for medications that are not found in the initial list found on page 6 (also shown below).

Please select all that apply ✱ *Required*

- ☐ L-dopa (immediate, controlled and dispersible)
- ☐ COMT-I
- ☐ Amantadine
- ☐ Dopamine Agonist
- ☐ MAOB-I
- ☐ Anticholinergic
- ☐ Advanced Therapies
- ☐ Other Medication (not listed)
- ☐ Not Applicable

Co-morbidities

This part of the survey uses a table of questions, view as separate questions instead?

Please select as applicable (existing prior to admission and ongoing) *Further information can be found by expanding the "more info" section.*

More info

	✱ *Required*		
	Yes	No	Unknown
Asthma	○	○	○
Chronic pulmonary disease	○	○	○
Diabetes Type 1	○	○	○
Diabetes Type 2	○	○	○
Dementia	○	○	○
Chronic neurological disorder (other than Parkinsonian syndromes)	○	○	○
Hypertension	○	○	○
Chronic cardiac disease (not hypertension)	○	○	○
Chronic kidney disease	○	○	○
Obesity	○	○	○
Moderate or severe liver disease	○	○	○
Mild liver disease	○	○	○
Asplenia	○	○	○
Malignant neoplasm	○	○	○

Please Note: This page is continued overleaf

Please note: The more info button expands to show:

Less info

CO-MORBIDITIES AND RISK FACTORS

In general, do not include past comorbidities that are no longer ongoing.

Where example conditions are given, these are not intended to be exhaustive and other conditions of equivalent severity should be included.

Chronic cardiac disease (not hypertension). Please include any of coronary artery disease, heart failure, congenital heart disease, cardiomyopathy, rheumatic heart disease. Hypertension Elevated arterial blood pressure diagnosed clinically, >140mmHg systolic or >90mmHg diastolic.

Chronic pulmonary disease. Please include any of chronic obstructive pulmonary disease (chronic bronchitis, chronic obstructive pulmonary disease (COPD), emphysema), cystic fibrosis, bronchiectasis, interstitial lung disease, pre-existing requirement for long term oxygen therapy.

Asthma (physician diagnosed).

Chronic Kidney Disease Please include any of clinician-diagnosed chronic kidney disease, chronic estimated glomerular filtration rate < 60 mL/min/1.73m2 , history of kidney transplantation.

Obesity (as defined by clinical staff) ideally but not necessarily with an objective measurement of obesity, such as calculation of the body mass index (BMI of 30 or more)

Moderate or severe liver disease. This is defined as cirrhosis with portal hypertension, with or without bleeding or a history of variceal bleeding.

Mild liver disease. This is defined as cirrhosis without portal hypertension or chronic hepatitis Asplenia Please include any of splenectomy, non-functional spleen, and congenital asplenia.

Chronic neurological disorder. Please include any of cerebral palsy, multiple sclerosis, motor neurone disease, muscular dystrophy, myasthenia gravis, stroke, severe learning difficulty.

Malignant neoplasm. Current solid organ or haematological malignancy. Please do not include malignancies that have been declared 'cured' ≥5 years ago with no evidence of ongoing disease. Do not include non-melanoma skin cancers. Do not include benign growths or dysplasia.

Chronic hematological disease. Any long-term disorder of the red or white blood cells, platelets or coagulation system requiring regular or intermittent treatment. Do not include leukaemia, lymphoma or myeloma, which should be entered under malignancy. Do not include iron-deficiency anaemia which is explained by diet or chronic blood loss.

Please Note: This page is continued overleaf

Please Note: This page is a continuation of the previous form

Chronic haematological disease	○	○	○
AIDS/HIV	○	○	○
Rheumatological disorder	○	○	○
TB	○	○	○
Malnutrition	○	○	○
Current smoker	○	○	○
Former smoker	○	○	○
Other (if other, please describe in free text below)	○	○	○

If other; please specify

Your answer should be no more than 50 characters long.

Has the individual received a MHRA approved COVID-19 vaccination? ✱ *Required*

○ Yes
○ No

Which vaccination did the individual receive? ❶ Required

○ Pfizer/BioNTech
○ Oxford/AstraZeneca

Please add vaccination dates

	Day ✱ *Required*	Month ✱ *Required*	Year 2020	Year 2021
First	Please select	Please select	○	○
Second	Please select	Please select	○	○

Please Note: This page is continuation of the previous form

AIDS/HIV. History of laboratory-confirmed HIV infection.

Diabetes Mellitus Type 1 or Type 2 diabetes mellitus requiring oral or subcutaneous treatment.

Rheumatological disorder. This is defined as an inflammatory and degenerative diseases of connective tissue structures. It includes chronic arthropathies and arthritis, connective tissue disorders and vasculitides.

Dementia. This is defined as clinical diagnosis of dementia.

Tuberculosis (TB). Patients currently receiving treatment for tuberculosis. Do not include latent tuberculosis.

Malnutrition. Any clinically identified deficiency in intake, either of total energy or of specific nutrients that led to a dietetic intervention or referral prior to the onset of COVID-19 symptoms. Do not include people who needed supplementary nutrition solely due to reduced intake during their current illness episode.

Current Smoker. Smoking at least one cigarette, cigar, pipe or equivalent per day before the onset of the current illness. Do not include smoke-free tobacco products such as chewed tobacco or electronic nicotine delivery devices.

Other relevant risk factor. List any significant risk factors or comorbidities that existed prior to admission, are ongoing, that are not already listed.

Please note: If yes is selected two new questions will appear asking for the correct vaccination to be selected and dates associated.

Please note: date box will automatically appear below vaccination choice.

Will drop down selection of dates (1-31) and also UNK and N/A. Please only select N/A if there is no second vaccination.

Will drop down selection of months (Jan-Dec) and also UNK and N/A. Please only select N/A if there is no second vaccination.

Page 6: COVID-19 Admission

Date of admission ✱ *Required*

Dates need to be in the format 'DD/MM/YYYY', for example 27/03/1980.

Please make sure the date is between 01/01/2020 and 30/06/2021.

(dd/mm/yyyy)

Date of COVID-19 positive test (date sample was taken) ✱ *Required*

Dates need to be in the format 'DD/MM/YYYY', for example 27/03/1980.

Please make sure the date is between 30/01/2020 and 30/06/2021.

(dd/mm/yyyy)

This part of the survey uses a table of questions, view as separate questions instead?

Reason for admission

	✱ *Required* Yes	No	Description of symptoms *Optional*
COVID-19 related symptoms	○	○	
Other	○	○	

Please note: Whilst Description of symptoms is optional—please endeavour to provide some information. Due to programming restrictions it was not possible to make this a required field.

Please note: It is possible to select both "COVID-19 related symptoms" and "Other". The choice of selecting one or both options is a clinical judgement.

Highest level of care provided ✱ *Required*

Please select ⌄

- Ward
- HDU
- ICU

Please Note: This page is continued overleaf

Please Note: This page is a continuation of the previous form

Please enter the date for admission into HDU/ICU **Required**

(dd/mm/yyyy)

Please enter the date for discharge from HDU/ICU **Required**

Dates need to be in the format 'DD/MM/YYYY', for example 27/03/1980.

(dd/mm/yyyy)

Severity of respiratory COVID-19 at worst * *Required*

Please select

- No evidence of infection (asymptomatic)
- Mild COVID symptoms not requiring additional support or therapy
- Ventilatory support required

Type of ventilatory support (maximum level used) **Required**

Please select

Please note: Only if ventilatory support selected is this applicable.

- Oxygen supplementation
- CPAP
- NIV
- Intubation

Did the patient have delirium associated with COVID-19 infection?

- Yes
- No

If yes, please select type **Required**

More info

- Hyperactive
- Hypoactive
- mixed

Hyperactive delirium is characterised by increased motor activity, restlessness, agitation, aggression, wandering, hyper alertness, hallucinations and delusions, and inappropriate behaviour.

Hypoactive delirium is characterised by reduced motor activity, lethargy, withdrawal, drowsiness and staring into space. It is the most common delirium in older people.

'Mixed' delirium is where people have features of hyperactive and hypoactive delirium.

Was the patient enrolled in a therapeutic COVID-19 clinical trial? **Required**

- Yes
- No

If yes, please describe

Your answer should be no more than 50 characters long.

UNIVERSITY OF PLYMOUTH

Outcome

Outcome of this admission ✱ *Required*

Please select

Discharged (choose location)
Still in same hospital
Discharged—end of life care
Died

The following questions are related to "Discharged (choose location).

Date of discharge ❗ **Required**

Dates need to be in the format 'DD/MM/YYYY', for example 27/03/1980.
Please make sure the date is between 01/03/2020 and 30/06/2021.

(dd/mm/yyyy)

Destination at discharge ❗ **Required**

Please select

Own home/private residence
Residential or nursing home
Local/community hospital
Hospice
Other

If you selected Other, please specify: ❗ **Required**

Is there a plan for ongoing rehabilitation at this location? ❗ **Required**

- ○ Yes
- ○ No

Please Note: This page is continued overleaf

Please Note: This page is a continuation of the previous form

Are the anti-Parkinson's medications the same at discharge as those on admission? **Required**

Please select

Yes
No
Unknown

If no, please enter all anti-Parkinsonian medications at discharge. Select all that apply. **Required**

- L-dopa (immediate, controlled and dispersible)
- COMT-I
- Amantadine
- Dopamine Agonist
- MAOB-I
- Anticholinergic
- Advanced Therapies
- Other
- Not Applicable

Please note: If "No" is selected then the medication pages will open following the same format as found on **Page 4— Parkinson's Medication on Admission found on pages 6-10 of this document.**

Please be aware that the programming limitation on "not applicable still applies"

The following questions are related to "Still in same hospital".

Location **Required**

Please select

Ward
HDU
ICU

Is there a plan for ongoing rehabilitation at this location? **Required**

- Yes
- No

Please Note: This page is continued overleaf

Please Note: This page is a continuation of the previous form

Date of discharge **Required**

Dates need to be in the format 'DD/MM/YYYY', for example 27/03/1980.
Please make sure the date is between 01/03/2020 and 30/06/2021.

(dd/mm/yyyy)

The following questions are related to "Discharged—end of life care".

Destination at discharge **Required**

Please select

Own home/private residence
Residential or nursing home
Local/community hospital
Hospice
Other

If you selected Other, please specify: **Required**

Date of death (week ending)
(Please enter the Sunday date of the week the individual died, e.g. if they died on 24th April 2020 - please enter 26/04/2020)

(dd/mm/yyyy)

A week is defined as Monday-Sunday.

When entering the date of death please ensure the date chosen is the Sunday of the week they died.

Cause of death

Please select

COVID-19
Other

Please Note: This page is continued overleaf

Please Note: This page is a continuation of the previous form

Please indicate how COVID-19 related cause of death information was gained

- ○ From death certificate
- ○ Based on clinical opinion

If you selected Other, please specify:

[ ]

Are the anti-Parkinson's medications the same at discharge as those on admission?

[No ▾]

If no, please enter all anti-Parkinsonian medications at discharge. Select all that apply.

Required

- ☐ L-dopa (immediate, controlled and dispersible)
- ☐ COMT-I
- ☐ Amantadine
- ☐ Dopamine Agonist
- ☐ MAOB-I
- ☐ Anticholinergic
- ☐ Advanced Therapies
- ☐ Other
- ☐ Not Applicable

Please note: If "No" is selected then the medication pages will open following the same format as found on **Page 4— Parkinson's Medication on Admission found on pages 6-10 of this document.**

Please be aware that the programming limitation on "not applicable still applies"

If Yes or Unknown is selected no further documentation of medicines are required.

Please Note: This page is continued overleaf

Please Note: This page is a continuation of the previous form

The following questions are related to "Died".

Date of death (week ending)
(Please enter the Sunday date of the week the individual died, e.g. if they died on 24th April 2020 - please enter 26/04/2020) Required

(dd/mm/yyyy)

A week is defined as Monday-Sunday. When entering the date of death please ensure the date chosen is the Sunday of the week they died.

Cause of death Required

Please select

COVID-19
Other

Please indicate how COVID-19 related cause of death information was gained

- From death certificate
- Based on clinical opinion

If you selected Other, please specify:

Final page

Thank you for entering this information. If you have spotted any errors in the survey or wish to confirm any information please contact jemma.inches@plymouth.ac.uk.

Please restart the questionnaire by clicking here https://plymouth.onlinesurveys.ac.uk/covid-19-and-pd-uk-survey-twc to add another individual's details.

Please note: This link is an example.

The link that will be in the survey will the link for the registered site's unique link. You will need to enter the password again and reconfirm consent to continue.

If the link is incorrect or does not work please contact the COVID 19 PD UK study team.
