# Supplementary material for: Clinical features and outcomes of hospitalised patients with COVID-19 and Parkinsonian disorders: A multicentre UK-based study
Source: PLoS One. 2023 Jul 31;18(7):e0285349. doi: 10.1371/journal.pone.0285349 (PMC10389727; doi:10.1371/journal.pone.0285349)
Supplement: S1 Checklist — (DOCX) [file pone.0285349.s002.docx]

STROBE Statement—checklist of items that should be included in reports of observational studies

|  | Item No. | Recommendation | Page  No. | Relevant text from manuscript |
| --- | --- | --- | --- | --- |
| **Title and abstract** | 1 | (*a*) Indicate the study’s design with a commonly used term in the title or the abstract | 3 | Study design is indicated in the Methods section of the abstract:  “A retrospective cohort study of Parkinson’s disease patients with a positive SARS-CoV-2 test admitted to hospital between February 2020 and July 2021.” |
|  |  | (*b*) Provide in the abstract an informative and balanced summary of what was done and what was found | 3 | This information is stated in the abstract: Objective, Methods and Results sections. |
| Introduction | | | |  |
| Background/rationale | 2 | Explain the scientific background and rationale for the investigation being reported | 5 | Scientific background and rationale for this study are reported in the Introduction section. |
| Objectives | 3 | State specific objectives, including any prespecified hypotheses | 5 | The final sentence of the Introduction section details the aims of the study:  “The COVID-19 PD UK study is the first to investigate the association of demographic, co-morbidity, COVID-19 and PD-specific factors with mortality and severe outcomes of PwP who tested positive for COVID-19 admitted to a UK NHS trust hospital.”  We do not state any hypotheses. |
| Methods | | | |  |
| Study design | 4 | Present key elements of study design early in the paper | 6 | The study design is described in the Methods section.  “The COVID-PD UK study was a retrospective cohort study across 21 acute care settings in England funded by Parkinson’s UK (Ref: G-2001).”  “The study consisted of patients with a clinical diagnosis of PD, APS (including progressive supranuclear palsy, multiple system atrophy) or Parkinson’s dementia syndrome (Parkinson’s disease dementia (PDD) or dementia with Lewy bodies (DLB)) admitted to participating hospitals between 5^th^ February 2020 and 31^st^ July 2021, with a positive polymerase chain reaction (PCR) test.”  “Clinical care teams completed an online survey (S3 Supporting information) on JISC (https://www.onlinesurveys.ac.uk/) using patients’ clinical care records, extracting information from admission to at least 28 days following admission. Retrospective data collection began in February 2021 and the online survey closed on the 31st July 2021. Individuals were pseudo-anonymised by sites to allow for data clarification where required.” |
| Setting | 5 | Describe the setting, locations, and relevant dates, including periods of recruitment, exposure, follow-up, and data collection | 6 | The setting, location and dates are included in the Methods section:  “The COVID-PD UK study was a retrospective cohort study across 21 acute care settings in England.”  “…admitted to participating hospitals between 5^th^ February 2020 and 31^st^ July 2021”  As this study is retrospective, there was no follow-up, data collection was performed for participants at least 28-days post-discharge:  “Clinical care teams completed an online survey (S3 Supporting information) on JISC (https://www.onlinesurveys.ac.uk/) using patients’ clinical care records, extracting information from admission to at least 28 days following admission.” |
| Participants | 6 | (*a*) *Cohort study*—Give the eligibility criteria, and the sources and methods of selection of participants. Describe methods of follow-up  *Case-control study*—Give the eligibility criteria, and the sources and methods of case ascertainment and control selection. Give the rationale for the choice of cases and controls  *Cross-sectional study*—Give the eligibility criteria, and the sources and methods of selection of participants | 6, 9 | The eligibility criteria is provided in the Methods section (paragraph two):  “The study consisted of patients with a clinical diagnosis of PD, APS (including progressive supranuclear palsy, multiple system atrophy) or Parkinson’s dementia syndrome (Parkinson’s disease dementia (PDD) or dementia with Lewy bodies (DLB)) admitted to participating hospitals between 5th February 2020 and 31st July 2021, with a positive polymerase chain reaction (PCR) test. During this time, NHS PCR testing was to some degree heterogeneous, details of testing methodologies and implementation during the pandemic are summarised by the UK Health Security Agency [20]. Exclusion criteria were patients with a diagnosis of vascular parkinsonism and a COVID-19 positive test over 2 weeks prior to admission or at any time following discharge.”  Participant selection was completed by sites and described in the Results section (paragraph 1):  “Six sites entered data comprehensively over waves one and two (n=242), while due to resource constraints, the other sites submitted data for one of the two waves or entered data from a selection of patients.” |
|  |  | (*b*) *Cohort study*—For matched studies, give matching criteria and number of exposed and unexposed  *Case-control study*—For matched studies, give matching criteria and the number of controls per case |  | NA |
| Variables | 7 | Clearly define all outcomes, exposures, predictors, potential confounders, and effect modifiers. Give diagnostic criteria, if applicable | 6, 7 | The outcome variables are described in the Methods section, paragraph 9.  “The primary outcome of the study was death from any cause within 28-days of a COVID-19 positive test. Date of death was recorded as the Sunday following death to preserve anonymity. We therefore used death within 34-days as a proxy outcome; this assumption was explored in a sensitivity analysis. Secondary outcomes included the requirement for respiratory support (oxygen supplementation, continuous positive airway pressure, non-invasive ventilation or intubation), an increase in the level of care post-discharge and change in levodopa equivalent daily dose (LEDD).”  Other variables include patient, Parkinson’s disease and COVID-19 characteristics, these are listed in the Supporting Information (S1) and non-standard variables are described in the Methods section:  “Data included patient, PD and COVID-19 related characteristics along with details of admission, discharge and participation in a COVID-19 related clinical trial. Comorbidities were chosen to allow comparison with the International Severe Acute Respiratory and emerging Infection Consortium (ISARIC) study of hospitalised COVID-19 patients in the UK [21]. Patient characteristics included age, sex, ethnicity, index of multiple deprivation (IMD), location pre-admission and clinical frailty score (CFS) [22]. PD related features captured included those considered neurological risk features in UK national guidance [23], such as significant cognitive impairment or psychosis, bulbar symptoms, significant respiratory compromise, significant autonomic neuropathy, as well as marked motor fluctuations and Hoehn and Yahr (H&Y) stage. PD features and comorbidities could be entered as unknown by site staff on the JISC survey.”  “The wave of positive SARS-CoV-2 test was identified. Wave one: 23/03/2020 to 30/05/2020, corresponding to wild-type SARS-CoV-2, and wave two: 07/09/2020 to 22/05/2021 [24] corresponding to the emergence of alpha and delta variants in the UK [25].”  “Patients were classified as vaccinated if their positive SARS-CoV-2 test was over 14 days following their first vaccine dose. COVID-19 was classified as hospital-acquired if the positive SARS-CoV-2 test was more than 5 days following admission; otherwise, COVID-19 was classified as community-acquired.”  “COVID-19 and non-COVID-19 symptoms at admission were recorded as free text and subsequently grouped by the research team into categories including altered mental state (delirium, confusion, obtundation, reduced oral intake), COVID-19 (pyrexia, cough, anosmia) and other respiratory symptoms. Symptoms were summarised for the community- and hospital-acquired COVID-19 patients separately.” |
| Data sources/ measurement | 8* | For each variable of interest, give sources of data and details of methods of assessment (measurement). Describe comparability of assessment methods if there is more than one group | 6, 7 | All data was collected from patient records:  “Clinical care teams completed an online survey (S3 Supporting information) on JISC (https://www.onlinesurveys.ac.uk/) using patients’ clinical care records, extracting information from admission to at least 28 days following admission. Retrospective data collection began in February 2021 and the online survey closed on the 31st July 2021. Individuals were pseudo-anonymised by sites to allow for data clarification where required.”  There was a querying process to identify unexpected responses which were returned to sites:  “No imputation of missing data was performed, however, sites were asked to address unexpected responses and missing data during the querying process by checking patient care records and providing the study team with updated responses where necessary.” |
| Bias | 9 | Describe any efforts to address potential sources of bias | 8 | We identified participants with unexpected and missing responses to query with sites:  “… sites were asked to address unexpected responses and missing data during the querying process by checking patient care records and providing the study team with updated responses where necessary.”  We conducted sensitivity analyses to assess the effects of site’s identification of participants (comprehensive or selective), wave of COVID-19 and extension of including deaths within 28-34 days:  “Sensitivity analyses were conducted for all outcomes using individuals with a positive COVID-19 test in wave two, sites that comprehensively selected their patients to enter over waves one and two and for the survival outcome, censoring at 28-days.”  All outcomes are assessed with univariable and multivariable analysis. |
| Study size | 10 | Explain how the study size was arrived at | 9 | Sites were encouraged to include all patients that are eligible, with details in the Methods section. The sample is described in Table 1. |

Continued on next page

| Quantitative variables | 11 | Explain how quantitative variables were handled in the analyses. If applicable, describe which groupings were chosen and why | 6, 7, S2 Supporting material | Variables are described in the Methods section, and in S1 Supporting information in Table form. |
| --- | --- | --- | --- | --- |
| Statistical methods | 12 | (*a*) Describe all statistical methods, including those used to control for confounding | 8 | All statistical methods are described in the Methods section:  “Survival within 34-days of a COVID-19 positive test was analysed using univariable and multivariable Cox proportional hazards models, using Schoenfeld’s test to assess the assumption of proportional hazards. The requirement for respiratory support and an increase in care were modelled using univariable and multivariable logistic regression models. Model selection for the final multivariable models was conducted using backward elimination, whilst including variables of clinical interest: sex, age, ethnicity, diagnosis, wave of COVID-19 positive test and where COVID-19 was acquired, regardless of their statistical significance. For each outcome, the first-order interaction of wave and where COVID-19 was acquired was examined for statistical significance. In the multivariable models, site was included as a random effect to account for potential differences in practice and COVID-19 severity between UK regions during the pandemic.” |
|  |  | (*b*) Describe any methods used to examine subgroups and interactions | 8 | The first order interaction of wave and where COVID-19 was acquired was examined:  “For each outcome, the first-order interaction of wave and where COVID-19 was acquired was examined for statistical significance. In the multivariable models, site was included as a random effect to account for potential differences in practice and COVID-19 severity between UK regions during the pandemic.”  “Sensitivity analyses were conducted for all outcomes using individuals with a positive COVID-19 test in wave two, sites that comprehensively selected their patients to enter over waves one and two and for the survival outcome, censoring at 28-days.” |
|  |  | (*c*) Explain how missing data were addressed | 7 | Described in the Methods section:  “ No imputation of missing data was performed, however, sites were asked to address unexpected responses and missing data during the querying process by checking patient care records and providing the study team with updated responses where necessary.” |
|  |  | (*d*) *Cohort study*—If applicable, explain how loss to follow-up was addressed  *Case-control study*—If applicable, explain how matching of cases and controls was addressed  *Cross-sectional study*—If applicable, describe analytical methods taking account of sampling strategy |  | NA |
|  |  | (*e*) Describe any sensitivity analyses | 8 | Sensitivity analyses were conducted and are described in the Methods section:  “Sensitivity analyses were conducted for all outcomes using individuals with a positive COVID-19 test in wave two, sites that comprehensively selected their patients to enter over waves one and two and for the survival outcome, censoring at 28-days.” |
| Results | | | | |
| Participants | 13* | (a) Report numbers of individuals at each stage of study—eg numbers potentially eligible, examined for eligibility, confirmed eligible, included in the study, completing follow-up, and analysed | 9 | This is described in the first paragraph of the Results section:  “Site staff from 21 hospitals entered 627 individual data sets; of these 17 were duplicates, 38 were excluded due to unverifiable data and 20 were excluded due to meeting the exclusion criteria. The final number of individual admission data sets included in the COVID-19 PD study was 552 compromising 385 community-acquired and 167 hospital-acquired COVID-19 infection episodes; the timing of these admissions is shown in Fig 1.” |
|  |  | (b) Give reasons for non-participation at each stage | 9 | As above. Non-participation reasons included duplicates, unverifiable data and eligibility. |
|  |  | (c) Consider use of a flow diagram |  | This was not deemed appropriated. |
| Descriptive data | 14* | (a) Give characteristics of study participants (eg demographic, clinical, social) and information on exposures and potential confounders | 9 | Table 1 and Supporting Information S1 detail participant characteristics. |
|  |  | (b) Indicate number of participants with missing data for each variable of interest | 9, S2 | Table 1 and Supporting information S1 provide total numbers of participants providing data for each variable of interest. |
|  |  | (c) *Cohort study*—Summarise follow-up time (eg, average and total amount) |  | This study is retrospective, so there is no follow-up time. All participants were discharged for at least 28 days prior to data collection. |
| Outcome data | 15* | *Cohort study*—Report numbers of outcome events or summary measures over time | 13, 14 | Numbers of participants reporting the outcomes of interest are detailed under each corresponding heading in the Results section:  “Of the 552 patients in the COVID-19 PD study, 212 (38.4%) died within 34-days of a COVID-19 positive test.”  “Over half of patients received respiratory support while in hospital (291/552, 52.7%)”  “Of these 264 patients, 115 individuals (43.6%) had an increase in care and 149 (56.4%) remained at their pre-admission location at discharge.” |
|  |  | *Case-control study—*Report numbers in each exposure category, or summary measures of exposure |  | NA |
|  |  | *Cross-sectional study—*Report numbers of outcome events or summary measures |  | NA |
| Main results | 16 | (*a*) Give unadjusted estimates and, if applicable, confounder-adjusted estimates and their precision (eg, 95% confidence interval). Make clear which confounders were adjusted for and why they were included | 13, 14, 15, S3 | Results from the three main models for the outcomes listed above are provided in Figs 5, 6 and 7.  Further details including univariable, multivariable and sensitivity analyses are listed in the Supporting information S2. |
|  |  | (*b*) Report category boundaries when continuous variables were categorized |  | NA |
|  |  | (*c*) If relevant, consider translating estimates of relative risk into absolute risk for a meaningful time period |  | NA |

Continued on next page

| Other analyses | 17 | Report other analyses done—eg analyses of subgroups and interactions, and sensitivity analyses | S3 | Sensitivity analyses are reported for each outcome in Supporting information S2. |
| --- | --- | --- | --- | --- |
| Discussion | | | | |
| Key results | 18 | Summarise key results with reference to study objectives | 16 | Rey results are summarised in the first paragraph of the Discussion. |
| Limitations | 19 | Discuss limitations of the study, taking into account sources of potential bias or imprecision. Discuss both direction and magnitude of any potential bias | 19 | Limitations are discussed in the final paragraph of the Discussion. |
| Interpretation | 20 | Give a cautious overall interpretation of results considering objectives, limitations, multiplicity of analyses, results from similar studies, and other relevant evidence | 19 | Limitations and references are discussed in the Discussion section. |
| Generalisability | 21 | Discuss the generalisability (external validity) of the study results | 16-19 | The study is referred to as:  “multicentre UK-based study”, the results are compared to other comparable countries during the pandemic. |
| Other information | |  | | |
| Funding | 22 | Give the source of funding and the role of the funders for the present study and, if applicable, for the original study on which the present article is based | 20 | The study funders are mentioned in the Methods section paragraph 1 and detail of their involvement is provided during the submission process. |

*Give information separately for cases and controls in case-control studies and, if applicable, for exposed and unexposed groups in cohort and cross-sectional studies.

**Note:** An Explanation and Elaboration article discusses each checklist item and gives methodological background and published examples of transparent reporting. The STROBE checklist is best used in conjunction with this article (freely available on the Web sites of PLoS Medicine at http://www.plosmedicine.org/, Annals of Internal Medicine at http://www.annals.org/, and Epidemiology at http://www.epidem.com/). Information on the STROBE Initiative is available at www.strobe-statement.org.
