# Supplementary material for: Clinical features and outcomes of hospitalised patients with COVID-19 and Parkinsonian disorders: A multicentre UK-based study
Source: PLoS One. 2023 Jul 31;18(7):e0285349. doi: 10.1371/journal.pone.0285349 (PMC10389727; doi:10.1371/journal.pone.0285349)
Supplement: S1 Table — (DOCX) [file pone.0285349.s003.docx]

**S1 Table: Summary of patient, Parkinson’s and COVID-19 characteristics by diagnosis.**

|  | | **Parkinson’s disease** | **Parkinson’s dementia syndrome** | **Atypical parkinsonian syndrome** | **Total** |
| --- | --- | --- | --- | --- | --- |
| **Ethnicity** | N | 349 | 170 | 33 | 552 |
| Any other ethnic group | N (%) | 9 (2.6) | 6 (3.5) | 1 (3.0) | 16 (2.9) |
| Arab |  | 2 (0.6) | 1 (0.6) | 0 (0.0) | 3 (0.5) |
| Asian / Asian British - Any other Asian background |  | 4 (1.1) | 1 (0.6) | 1 (3.0) | 6 (1.1) |
| Asian / Asian British - Bangladeshi |  | 1 (0.3) | 0 (0.0) | 0 (0.0) | 1 (0.2) |
| Asian / Asian British - Indian |  | 10 (2.9) | 3 (1.8) | 2 (6.1) | 15 (2.7) |
| Asian / Asian British - Pakistani |  | 5 (1.4) | 1 (0.6) | 0 (0.0) | 6 (1.1) |
| Black or Black British - African |  | 1 (0.3) | 0 (0.0) | 1 (3.0) | 2 (0.4) |
| Black or Black British - Caribbean |  | 3 (0.9) | 2 (1.2) | 0 (0.0) | 5 (0.9) |
| Chinese |  | 1 (0.3) | 0 (0.0) | 1 (3.0) | 2 (0.4) |
| Mixed Race - Any other mixed / Multiple Ethnic background |  | 0 (0.0) | 1 (0.6) | 0 (0.0) | 1 (0.2) |
| Mixed Race - White and Asian |  | 1 (0.3) | 0 (0.0) | 0 (0.0) | 1 (0.2) |
| Mixed Race - White and Black African |  | 1 (0.3) | 1 (0.6) | 0 (0.0) | 2 (0.4) |
| Mixed Race - White and Black Caribbean |  | 1 (0.3) | 0 (0.0) | 0 (0.0) | 1 (0.2) |
| White - Any other White background |  | 10 (2.9) | 2 (1.2) | 1 (3.0) | 13 (2.4) |
| White - English / Welsh / Scottish / Northern Irish / British |  | 298 (85.4) | 152 (89.4) | 26 (78.8) | 476 (86.2) |
| White - Irish |  | 2 (0.6) | 0 (0.0) | 0 (0.0) | 2 (0.4) |
| **IMD decile** | N | 349 | 170 | 33 | 552 |
| 1 | N (%) | 32 (9.2) | 13 (7.6) | 4 (12.1) | 49 (8.9) |
| 2 |  | 31 (8.9) | 15 (8.8) | 1 (3.0) | 47 (8.5) |
| 3 |  | 42 (12.0) | 9 (5.3) | 2 (6.1) | 53 (9.6) |
| 4 |  | 35 (10.0) | 14 (8.2) | 2 (6.1) | 51 (9.2) |
| 5 |  | 38 (10.9) | 17 (10.0) | 6 (18.2) | 61 (11.1) |
| 6 |  | 39 (11.2) | 20 (11.8) | 3 (9.1) | 62 (11.2) |
| 7 |  | 28 (8.0) | 25 (14.7) | 5 (15.2) | 58 (10.5) |
| 8 |  | 41 (11.7) | 13 (7.6) | 3 (9.1) | 57 (10.3) |
| 9 |  | 29 (8.3) | 27 (15.9) | 4 (12.1) | 60 (10.9) |
| 10 |  | 34 (9.7) | 17 (10.0) | 3 (9.1) | 54 (9.8) |
| **Asthma** | N | 341 | 164 | 33 | 538 |
| No | N (%) | 304 (89.1) | 149 (90.9) | 31 (93.9) | 484 (90.0) |
| Yes |  | 37 (10.9) | 15 (9.1) | 2 (6.1) | 54 (10.0) |
| **Chronic pulmonary disease** | N | 335 | 164 | 33 | 532 |
| No | N (%) | 291 (86.9) | 145 (88.4) | 30 (90.9) | 466 (87.6) |
| Yes |  | 44 (13.1) | 19 (11.6) | 3 (9.1) | 66 (12.4) |
| **Diabetes** | N | 344 | 168 | 33 | 545 |
| No | N (%) | 270 (78.5) | 138 (82.1) | 26 (78.8) | 434 (79.6) |
| Yes |  | 74 (21.5) | 30 (17.9) | 7 (21.2) | 111 (20.4) |
| **Dementia** | N | 333 | 170 | 32 | 535 |
| No | N (%) | 289 (86.8) | 0 (0.0) | 28 (87.5) | 317 (59.3) |
| Yes |  | 44 (13.2) | 170 (100.0) | 4 (12.5) | 218 (40.7) |
| **Chronic neurological disorder** | N | 339 | 163 | 33 | 535 |
| No | N (%) | 285 (84.1) | 142 (87.1) | 28 (84.8) | 455 (85.0) |
| Yes |  | 54 (15.9) | 21 (12.9) | 5 (15.2) | 80 (15.0) |
| **Hypertension** | N | 345 | 165 | 32 | 542 |
| No | N (%) | 184 (53.3) | 94 (57.0) | 13 (40.6) | 291 (53.7) |
| Yes |  | 161 (46.7) | 71 (43.0) | 19 (59.4) | 251 (46.3) |
| **Chronic cardiac disease** | N | 342 | 165 | 32 | 539 |
| No | N (%) | 216 (63.2) | 107 (64.8) | 23 (71.9) | 346 (64.2) |
| Yes |  | 126 (36.8) | 58 (35.2) | 9 (28.1) | 193 (35.8) |
| **Chronic kidney disease** | N | 343 | 160 | 32 | 535 |
| No | N (%) | 264 (77.0) | 129 (80.6) | 25 (78.1) | 418 (78.1) |
| Yes |  | 79 (23.0) | 31 (19.4) | 7 (21.9) | 117 (21.9) |
| **Obesity** | N | 325 | 153 | 32 | 510 |
| No | N (%) | 302 (92.9) | 149 (97.4) | 29 (90.6) | 480 (94.1) |
| Yes |  | 23 (7.1) | 4 (2.6) | 3 (9.4) | 30 (5.9) |
| **Liver disease** | N | 337 | 165 | 32 | 534 |
| No | N (%) | 329 (97.6) | 162 (98.2) | 30 (93.8) | 521 (97.6) |
| Yes |  | 8 (2.4) | 3 (1.8) | 2 (6.2) | 13 (2.4) |
| **Asplenia** | N | 343 | 169 | 33 | 545 |
| No | N (%) | 343 (100.0) | 169 (100.0) | 33 (100.0) | 545 (100.0) |
| **Malignant neoplasm** | N | 344 | 168 | 33 | 545 |
| No | N (%) | 307 (89.2) | 157 (93.5) | 31 (93.9) | 495 (90.8) |
| Yes |  | 37 (10.8) | 11 (6.5) | 2 (6.1) | 50 (9.2) |
| **Chronic haematological disease** | N | 335 | 170 | 32 | 537 |
| No | N (%) | 311 (92.8) | 167 (98.2) | 30 (93.8) | 508 (94.6) |
| Yes |  | 24 (7.2) | 3 (1.8) | 2 (6.2) | 29 (5.4) |
| **AIDS/HIV** | N | 348 | 170 | 33 | 551 |
| No | N (%) | 348 (100.0) | 170 (100.0) | 33 (100.0) | 551 (100.0) |
| **Rheumatological disorder** | N | 340 | 166 | 33 | 539 |
| No | N (%) | 277 (81.5) | 138 (83.1) | 25 (75.8) | 440 (81.6) |
| Yes |  | 63 (18.5) | 28 (16.9) | 8 (24.2) | 99 (18.4) |
| **TB** | N | 345 | 169 | 33 | 547 |
| No | N (%) | 341 (98.8) | 168 (99.4) | 33 (100.0) | 542 (99.1) |
| Yes |  | 4 (1.2) | 1 (0.6) | 0 (0.0) | 5 (0.9) |
| **Malnutrition** | N | 324 | 158 | 30 | 512 |
| No | N (%) | 306 (94.4) | 151 (95.6) | 28 (93.3) | 485 (94.7) |
| Yes |  | 18 (5.6) | 7 (4.4) | 2 (6.7) | 27 (5.3) |
| **History of smoking** | N | 201 | 88 | 22 | 311 |
| No | N (%) | 147 (73.1) | 63 (71.6) | 18 (81.8) | 228 (73.3) |
| Yes |  | 54 (26.9) | 25 (28.4) | 4 (18.2) | 83 (26.7) |
| **Significant cognitive impairment/psychosis** | N | 335 | 170 | 30 | 535 |
|  |  |  |  |  |  |
|  |  |  |  |  |  |
| No | N (%) | 258 (77.0) | 4 (2.4) | 22 (73.3) | 284 (53.1) |
| Yes |  | 77 (23.0) | 166 (97.6) | 8 (26.7) | 251 (46.9) |
| **Bulbar symptoms** | N | 331 | 160 | 32 | 523 |
| No | N (%) | 282 (85.2) | 127 (79.4) | 16 (50.0) | 425 (81.3) |
| Yes |  | 49 (14.8) | 33 (20.6) | 16 (50.0) | 98 (18.7) |
| **Significant respiratory compromise** | N | 345 | 163 | 31 | 539 |
| No | N (%) | 338 (98.0) | 160 (98.2) | 31 (100.0) | 529 (98.1) |
| Yes |  | 7 (2.0) | 3 (1.8) | 0 (0.0) | 10 (1.9) |
| **Significant autonomic neuropathy** | N | 323 | 157 | 30 | 510 |
| No | N (%) | 254 (78.6) | 125 (79.6) | 21 (70.0) | 400 (78.4) |
| Yes |  | 69 (21.4) | 32 (20.4) | 9 (30.0) | 110 (21.6) |
| **Marked motor fluctuations** | N | 305 | 157 | 31 | 493 |
| No | N (%) | 221 (72.5) | 96 (61.1) | 22 (71.0) | 339 (68.8) |
| Yes |  | 84 (27.5) | 61 (38.9) | 9 (29.0) | 154 (31.2) |
| **Vaccinated** | N | 306 | 142 | 29 | 477 |
| No | N (%) | 290 (94.8) | 134 (94.4) | 29 (100.0) | 453 (95.0) |
| Yes |  | 16 (5.2) | 8 (5.6) | 0 (0.0) | 24 (5.0) |
| **Severity of respiratory COVID-19** | N | 349 | 170 | 33 | 552 |
| Asymptomatic | N (%) | 59 (16.9) | 40 (23.5) | 6 (18.2) | 105 (19.0) |
| Mild symptoms |  | 109 (31.2) | 42 (24.7) | 5 (15.2) | 156 (28.3) |
| Respiratory support required |  | 181 (51.9) | 88 (51.8) | 22 (66.7) | 291 (52.7) |
| **Delirium** | N | 349 | 170 | 33 | 552 |
| None | N (%) | 235 (67.3) | 92 (54.1) | 26 (78.8) | 353 (63.9) |
| Mixed |  | 41 (11.7) | 23 (13.5) | 1 (3.0) | 65 (11.8) |
| Hypoactive |  | 48 (13.8) | 48 (28.2) | 4 (12.1) | 100 (18.1) |
| Hyperactive |  | 25 (7.2) | 7 (4.1) | 2 (6.1) | 34 (6.2) |
| **Wave of positive COVID-19 test** | N | 349 | 170 | 33 | 552 |
| Wave 1 | N (%) | 110 (31.5) | 71 (41.8) | 9 (27.3) | 190 (34.4) |
| Wave 2 |  | 227 (65.0) | 92 (54.1) | 24 (72.7) | 343 (62.1) |
| Other |  | 12 (3.4) | 7 (4.1) | 0 (0.0) | 19 (3.4) |
| **COVID-19 acquired** | N | 349 | 170 | 33 | 552 |
| Community | N (%) | 253 (72.5) | 108 (63.5) | 24 (72.7) | 385 (69.7) |
| Hospital |  | 96 (27.5) | 62 (36.5) | 9 (27.3) | 167 (30.3) |
| **Highest level of care** | N | 349 | 170 | 33 | 552 |
| HDU | N (%) | 3 (0.9) | 0 (0.0) | 0 (0.0) | 3 (0.5) |
| ICU |  | 5 (1.4) | 1 (0.6) | 0 (0.0) | 6 (1.1) |
| Ward |  | 341 (97.7) | 169 (99.4) | 33 (100.0) | 543 (98.4) |
| **Discharge destination** | N | 247 | 96 | 20 | 363 |
| Local/community hospital | N (%) | 45 (18.2) | 8 (8.3) | 3 (15.0) | 56 (15.4) |
| Own home/private residence |  | 111 (44.9) | 32 (33.3) | 11 (55.0) | 154 (42.4) |
| Residential or nursing home |  | 91 (36.8) | 56 (58.3) | 6 (30.0) | 153 (42.1) |
| **Rehabilitation at discharge location** | N | 240 | 81 | 19 | 340 |
| No | N (%) | 135 (56.2) | 56 (69.1) | 12 (63.2) | 203 (59.7) |
| Yes |  | 105 (43.8) | 25 (30.9) | 7 (36.8) | 137 (40.3) |
| **Medications the same at admission and discharge** | N | 246 | 96 | 20 | 362 |
| No | N (%) | 67 (27.2) | 29 (30.2) | 6 (30.0) | 102 (28.2) |
| Unknown |  | 1 (0.4) | 0 (0.0) | 0 (0.0) | 1 (0.3) |
| Yes |  | 178 (72.4) | 67 (69.8) | 14 (70.0) | 259 (71.5) |

Abbreviations: Index of multiple deprivation (IMD), acquired immune deficiency syndrome (AIDS), human immunodeficiency virus (HIV), Tuberculosis (TB), Levodopa equivalent daily dose (LEDD), high-dependency care unit (HDU), intensive care unit (ICU).
