# Supplementary material for: Clinical features and outcomes of hospitalised patients with COVID-19 and Parkinsonian disorders: A multicentre UK-based study
Source: PLoS One. 2023 Jul 31;18(7):e0285349. doi: 10.1371/journal.pone.0285349 (PMC10389727; doi:10.1371/journal.pone.0285349)
Supplement: S2 Table — (DOCX) [file pone.0285349.s004.docx]

**S2 Table: Time from positive COVID-19 test to discharge by patient characteristics**.

|  | **N** | **Median (IQR) time from positive COVID-19 test to discharge (days)** |
| --- | --- | --- |
| **Sex** |  |  |
| Male | 227 | 14 (6-27) |
| Female | 136 | 12 (7-20) |
| **Ethnicity** |  |  |
| White British | 313 | 13 (6-24) |
| Other | 50 | 15 (9-23) |
| **Significant cognitive impairment** |  |  |
| No | 206 | 14 (7-25) |
| Yes | 148 | 13 (6-23) |
| **Bulbar symptoms** |  |  |
| No | 293 | 13 (6-23) |
| Yes | 54 | 17 (8-27) |
| **Significant respiratory compromise** |  |  |
| No | 351 | 13 (6-25) |
| Yes | 5 | 10 (9-11) |
| **Significant autonomic neuropathy** |  |  |
| No | 268 | 13 (7-22) |
| Yes | 74 | 14 (6-31) |
| **Marked motor fluctuations** |  |  |
| No | 241 | 13 (5-25) |
| Yes | 90 | 13 (7-25) |
| **Clinical frailty score** |  |  |
| <5 | 65 | 12 (5-20) |
| 5-6 | 179 | 14 (7-25) |
| 7-9 | 115 | 12 (7-24) |
| **Hoehn and Yahr** |  |  |
| 1-2 | 42 | 15 (6-22) |
| 2.5-3 | 119 | 11 (5-25) |
| 4-5 | 182 | 14 (7-24) |
| **Vaccinated** |  |  |
| No | 293 | 14 (7-24) |
| Yes | 19 | 8 (3-19) |
| **Severity of respiratory COVID-19** |  |  |
| Asymptomatic | 94 | 12 (6-26) |
| Mild symptoms | 125 | 12 (6-23) |
| Respiratory support required | 144 | 14 (8-23) |
| **Delirium** |  |  |
| No | 245 | 12 (6-21) |
| Yes | 118 | 15 (7-29) |
| **Wave of positive COVID-19 test** |  |  |
| Wave 1 | 110 | 12 (7-19) |
| Wave 2 | 242 | 15 (6-27) |
| Other | 11 | 7 (3-13) |
| **COVID-19 acquired** |  |  |
| Community | 250 | 12 (7-21) |
| Hospital | 113 | 16 (5-28) |
| **Asthma** |  |  |
| No | 319 | 14 (7-25) |
| Yes | 36 | 10 (3-20) |
| **Chronic pulmonary disease** |  |  |
| No | 308 | 13 (7-25) |
| Yes | 43 | 11 (6-22) |
| **Diabetes** |  |  |
| No | 288 | 13 (6-24) |
| Yes | 73 | 11 (7-20) |
| **Dementia** |  |  |
| No | 230 | 13 (7-25) |
| Yes | 123 | 12 (6-21) |
| **Chronic neurological disorder** |  |  |
| No | 303 | 12 (6-22) |
| Yes | 52 | 19 (10-31) |
| **Hypertension** |  |  |
| No | 199 | 13 (6-25) |
| Yes | 160 | 13 (7-22) |
| **Chronic cardiac disease** |  |  |
| No | 238 | 13 (6-24) |
| Yes | 119 | 13 (7-23) |
| **Chronic kidney disease** |  |  |
| No | 283 | 13 (6-24) |
| Yes | 72 | 12 (7-22) |
| **Obesity** |  |  |
| No | 314 | 13 (7-23) |
| Yes | 23 | 9 (4-20) |
| **Liver disease** |  |  |
| No | 349 | 13 (6-24) |
| Yes | 6 | 12 (9-17) |
| **Malignant neoplasm** |  |  |
| No | 327 | 13 (6-23) |
| Yes | 31 | 14 (6-32) |
| **Chronic haematological disease** |  |  |
| No | 335 | 13 (6-24) |
| Yes | 18 | 15 (9-18) |
| **Rheumatological disorder** |  |  |
| No | 292 | 14 (7-25) |
| Yes | 64 | 10 (6-18) |
| **TB** |  |  |
| No | 357 | 13 (7-23) |
| Yes | 3 | 5 (4-30) |
| **Malnutrition** |  |  |
| No | 322 | 13 (6-24) |
| Yes | 16 | 14 (2-18) |
| **History of smoking** |  |  |
| No | 161 | 11 (5-21) |
| Yes | 52 | 13 (7-26) |

Excluding participants who have died in hospital or remained in hospital at the end of the observation period. Abbreviations: Interquartile range (IQR).
