# Supplementary material for: Clinical features and outcomes of hospitalised patients with COVID-19 and Parkinsonian disorders: A multicentre UK-based study
Source: PLoS One. 2023 Jul 31;18(7):e0285349. doi: 10.1371/journal.pone.0285349 (PMC10389727; doi:10.1371/journal.pone.0285349)
Supplement: S3 Table — (DOCX) [file pone.0285349.s005.docx]

**S3 Table:** **Univariable, multivariable and multivariable sensitivity analysis results from Cox proportional hazards models of mortality within 34 days of COVID-19 positive test.**

|  | | **Hazard Ratio**  **(95% CI, p-value)**  **Univariable** | **Hazard Ratio**  **(95% CI, p-value)**  **Multivariable** | **Hazard Ratio**  **(95% CI, p-value)**  **Multivariable, comprehensive sensitivity analysis** | **Hazard Ratio**  **(95% CI, p-value)**  **Multivariable, wave 2 sensitivity analysis** | **Hazard Ratio**  **(95% CI, p-value)**  **Multivariable, 28-day sensitivity analysis** |
| --- | --- | --- | --- | --- | --- | --- |
| **Acquired COVID-19** | Community | - | - | - | - | - |
|  | Hospital | 0.85 (0.63 to 1.15, 0.304) | 1.16 (0.82 to 1.64, 0.395) | 1.07 (0.65 to 1.74, 0.797) | 1.26 (0.79 to 2.01, 0.341) | 0.90 (0.33 to 2.49, 0.090) |
| **Age at admission** | | 1.03 (1.02 to 1.05, 0.574) | 1.05 (1.03 to 1.07, <0.001) | 1.05 (1.01 to 1.08, 0.005) | 1.06 (1.03 to 1.09, <0.001) | 1.05 (1.03 to 1.07, <0.001) |
| **Diagnosis** | Parkinson’s disease | - | - | - | - | - |
|  | Parkinson’s dementia syndrome | 1.90 (1.44 to 2.52, <0.001) | 1.59 (1.14 to 2.20, 0.006) | 1.95 (1.18 to 3.22, 0.009) | 1.76 (1.08 to 2.86, 0.024) | 1.47 (1.05 to 2.06, 0.026) |
|  | Atypical parkinsonian syndrome | 1.65 (0.96 to 2.83, 0.069) | 1.39 (0.74 to 2.63, 0.306) | 1.71 (0.77 to 3.78, 0.185) | 1.64 (0.71 to 3.83, 0.250) | 1.25 (0.65 to 2.43, 0.502) |
| **Ethnicity** | White British | - | - | - | - | - |
|  | Other | 1.07 (0.73 to 1.58, 0.729) | 1.24 (0.81 to 1.88, 0.327) | 0.92 (0.42 to 2.03, 0.836) | 1.17 (0.57 to 2.41, 0.662) | 1.23 (0.79 to 1.89, 0.357) |
| **Sex** | Male | - | - | - | - | - |
|  | Female | 0.65 (0.48 to 0.88, 0.005) | 0.54 (0.39 to 0.75, <0.001) | 0.77 (0.47 to 1.25, 0.288) | 0.69 (0.57 to 1.10, 0.120) | 0.55 (0.39 to 0.77, <0.001) |
| **Wave of COVID-19** | 1 | 1.65 (1.25 to 2.17, <0.001) | 1.24 (0.91 to 1.70, 0.171) | 1.55 (1.00 to 2.42, 0.052) |  | 1.05 (0.73 to 1.51, 0.172) |
|  | 2 | - | - | - |  | - |
|  | Other | 0.74 (0.30 to 1.81, 0.512) | 1.07 (0.43 to 2.65, 0.887) | 2.86 (0.65 to 12.59, 0.164) |  | 1.25 (0.91 to 1.72, 0.845) |
| **Clinical frailty score** | <5 | 0.29 (0.17 to 0.50, <0.001) | 0.62 (0.44 to 0.85, 0.004) | 0.73 (0.45 to 1.18, 0.916) | 0.46 (0.20 to 1.06, 0.068) | 0.60 (0.33 to 1.08, 0.090) |
|  | 5-6 | 0.56 (0.42 to 0.75, <0.001) | 0.59 (0.33 to 1.07, 0.085) | 0.96 (0.44 to 2.08, 0.200) | 0.43 (0.27 to 0.68, <0.001) | 0.60 (0.43 to 0.84, 0.003) |
|  | 7-9 | - | - | - | - | - |
| **Severity of respiratory COVID-19** | Asymptomatic | 0.25 (0.15 to 0.40, <0.001) | 0.22 (0.12 to 0.39, <0.001) | 0.24 (0.11 to 0.54, <0.001) | 0.25 (0.12 to 0.49, <0.001) | 0.23 (0.13 to 0.43, <0.001) |
|  | Mild | 0.33 (0.23 to 0.47, <0.001) | 0.27 (0.18 to 0.41, <0.001) | 0.28 (0.15 to 0.50, <0.001 | 0.22 (0.11 to 0.43, <0.001) | 0.24 (0.16 to 0.38, <0.001) |
|  | Respiratory support | - | - | - | - | - |
| **Vaccinated** | No | - | - | - | - | - |
|  | Yes | 0.37 (0.14 to 0.99, 0.048) | 0.36 (0.13 to 0.99, 0.048) | 0.10 (0.01 to 0.75, 0.025) | 0.39 (0.14 o 1.09, 0.072) | 0.37 (0.13 to 1.02, 0.055) |
| **Significant cognitive impairment** | No | - |  |  |  |  |
|  | Yes | 1.84 (1.39 to 2.44, <0.001) |  |  |  |  |
| **Bulbar symptoms** | No | - |  |  |  |  |
|  | Yes | 1.42 (1.02 to 1.98, 0.037) |  |  |  |  |
| **Significant respiratory compromise** | No | - |  |  |  |  |
|  | Yes | 2.13 (0.95 to 4.81, 0.067) |  |  |  |  |
| **Significant autonomic neuropathy** | No | - |  |  |  |  |
|  | Yes | 0.88 (0.62 to 1.27, 0.500) |  |  |  |  |
| **Marked motor fluctuations** | No | - |  |  |  |  |
|  | Yes | 1.38 (1.02 to 1.86, 0.036) |  |  |  |  |
| **PD duration** | | 1.01 (0.99 to 1.04, 0.329) |  |  |  |  |
| **Admission LEDD** | | 0.97 (0.93 to 1.01, 0.091) |  |  |  |  |
| **Hoehn and Yahr** | 1-2 | 0.39 (0.21 to 0.72, 0.003) |  |  |  |  |
|  | 2.5-3 | 0.71 (0.52 to 0.97, 0.029) |  |  |  |  |
|  | 4-5 | - |  |  |  |  |
| **IMD decile** | 1 | - |  |  |  |  |
|  | 2 | 1.04 (0.55 to 1.98, 0.902) |  |  |  |  |
|  | 3 | 0.77 (0.39 to 1.49, 0.433) |  |  |  |  |
|  | 4 | 1.07 (0.57 to 1.99, 0.832) |  |  |  |  |
|  | 5 | 1.03 (0.56 to 1.9, 0.912) |  |  |  |  |
|  | 6 | 1.02 (0.56 to 1.87, 0.938) |  |  |  |  |
|  | 7 | 1.25 (0.69 to 2.26, 0.461) |  |  |  |  |
|  | 8 | 1.22 (0.67 to 2.22, 0.507) |  |  |  |  |
|  | 9 | 0.98 (0.52 to 1.81, 0.937) |  |  |  |  |
|  | 10 | 0.91 (0.48 to 1.72, 0.777) |  |  |  |  |
| **Location pre-admission** | Own home/private residence | 0.67 (0.51 to 0.89, 0.006) |  |  |  |  |
|  | Residential care/nursing home/local or community hospital | - |  |  |  |  |
| **Asthma** | No | - |  |  |  |  |
|  | Yes | 0.96 (0.60 to 1.54, 0.858) |  |  |  |  |
| **Chronic pulmonary disease** | No | - |  |  |  |  |
|  | Yes | 0.99 (0.65 to 1.51, 0.974) |  |  |  |  |
| **Diabetes** | No | - |  |  |  |  |
|  | Yes | 0.95 (0.67 to 1.34, 0.758) |  |  |  |  |
| **Dementia** | No | - |  |  |  |  |
|  | Yes | 1.92 (1.46 to 2.52, <0.001) |  |  |  |  |
| **Chronic neurological disorder** | No | - |  |  |  |  |
|  | Yes | 1.00 (0.68 to 1.47, 0.992) |  |  |  |  |
| **Hypertension** | No | - |  |  |  |  |
|  | Yes | 1.13 (0.86 to 1.48, 0.391) |  |  |  |  |
| **Chronic cardiac disease** | No | - |  |  |  |  |
|  | Yes | 1.49 (1.13 to 1.96, 0.005) |  |  |  |  |
| **Chronic kidney disease** | No | - |  |  |  |  |
|  | Yes | 1.28 (0.93 to 1.76, 0.124) |  |  |  |  |
| **Obesity** | No | - |  |  |  |  |
|  | Yes | 0.63 (0.31 to 1.29, 0.206) |  |  |  |  |
| **Malignant neoplasm** | No | - |  |  |  |  |
|  | Yes | 0.98 (0.61 to 1.57, 0.924) |  |  |  |  |
| **Chronic haematological disease** | No | - |  |  |  |  |
|  | Yes | 1.07 (0.59 to 1.97, 0.817) |  |  |  |  |
| **Rheumatological disorder** | No | - |  |  |  |  |
|  | Yes | 1.01 (0.71 to 1.44, 0.953) |  |  |  |  |
| **Malnutrition** | No | - |  |  |  |  |
|  | Yes | 1.21 (0.68 to 2.17, 0.520) |  |  |  |  |
| **History of smoking** | No | - |  |  |  |  |
|  | Yes | 1.08 (0.72 to 1.64, 0.700) |  |  |  |  |
| **Delirium** | No | - |  |  |  |  |
|  | Yes | 1.31 (0.99 to 1.72, 0.055) |  |  |  |  |
| **Liver disease** | No | - |  |  |  |  |
|  | Yes | 1.62 (0.76 to 3.44, 0.210) |  |  |  |  |

Abbreviations: Levodopa equivalent daily dose (LEDD), Index of multiple deprivation (IMD).
