# Supplementary material for: Clinical features and outcomes of hospitalised patients with COVID-19 and Parkinsonian disorders: A multicentre UK-based study
Source: PLoS One. 2023 Jul 31;18(7):e0285349. doi: 10.1371/journal.pone.0285349 (PMC10389727; doi:10.1371/journal.pone.0285349)
Supplement: S4 Table — (DOCX) [file pone.0285349.s006.docx]

**S4 Table: Univariable, multivariable and sensitivity analyses from mixed effects logistic regression models of requiring respiratory support.**

|  | | **Odds ratio**  **(95% CI, p-value)**  **Univariable** | **Odds ratio**  **(95% CI, p-value)**  **Multivariable** | **Odds ratio**  **(95% CI, p-value)**  **Multivariable, comprehensive sensitivity analysis** | **Odds ratio**  **(95% CI, p-value)**  **Multivariable, wave 2 sensitivity analysis** |
| --- | --- | --- | --- | --- | --- |
| **Acquired COVID-19** | Community | - | - | - | - |
|  | Hospital | 0.55 (0.38 to 0.80, 0.002) | 0.54 (0.36 to 0.81, 0.003) | 0.55 (0.31 to 0.98, 0.042) | 0.47 (0.28 to 0.77, 0.003) |
| **Age at admission** | | 0.99 (0.97 to 1.01, 0.557) | 1.00 (0.98 to 1.02, 0.850) | 0.99 (0.96 to 1.03, 0.594) | 1.00 (0.98 to 1.03, 0.732) |
| **Diagnosis** | Parkinson’s disease | - | - | - | - |
|  | Parkinson’s dementia syndrome | 1.00 (0.69 to 1.44, 0.983) | 0.93 (0.61 to 1.41, 0.731) | 0.89 (0.49 to 1.60, 0.691) | 0.77 (0.44 to 1.33, 0.346) |
|  | Atypical parkinsonian syndrome | 1.86 (0.89 to 4.08, 0.108) | 1.88 (0.83 to 4.23, 0.128) | 2.03 (0.66 to 6.21, 0.217) | 2.72 (1.00 to 7.39, 0.049) |
| **Ethnicity** | White British | - | - | - | - |
|  | Other | 1.20 (0.74 to 1.96, 0.468) | 0.90 (0.51 to 1.61, 0.733) | 1.16 (0.45 to 3.01, 0.761) | 0.78 (0.36 to 1.70, 0.533) |
| **Sex** | Male | - | - | - | - |
|  | Female | 0.98 (0.69 to 1.40, 0.917) | 0.94 (0.64 to 1.39, 0.765) | 1.10 (0.60 to 2.01, 0.764) | 0.83 (0.51 to 1.36, 0.469) |
| **COVID-19 wave** | 1 | 1.77 (1.23 to 2.55, 0.002) | 1.50 (0.98 to 2.29, 0.060) | 1.33 (0.73 to 2.42, 0.357) |  |
|  | 2 | - | - | - |  |
|  | Other | 0.78 (0.30 to 1.99, 0.611) | 0.71 (0.26 to 1.98, 0.513) | 0.82 (0.16 to 4.10, 0.810) |  |
| **Chronic neurological disorder** | No | - | - | - | - |
|  | Yes | 1.87 (1.15 to 3.12, 0.014) | 1.81 (1.06 to 3.09, 0.0310 | 1.68 (0.81 to 3.51, 0.167) | 1.49 (0.75 to 2.96, 0.251) |
| **Clinical frailty score** | <5 | 0.66 (0.39 to 1.11, 0.116) |  |  |  |
|  | 5-6 | 0.88 (0.61 to 1.28, 0.513) |  |  |  |
|  | 7-9 | - |  |  |  |
| **Vaccinated** | No | - |  |  |  |
|  | Yes | 0.93 (0.41 to 2.15, 0.855) |  |  |  |
| **Significant cognitive impairment** | No | - |  |  |  |
|  | Yes | 1.21 (0.86 to 1.70, 0.280) |  |  |  |
| **Bulbar symptoms** | No | - |  |  |  |
|  | Yes | 1.05 (0.68 to 1.64, 0.817) |  |  |  |
| **Significant respiratory compromise** | No | - |  |  |  |
|  | Yes | 2.16 (0.59 to 10.08, 0.270) |  |  |  |
| **Significant autonomic neuropathy** | No | - |  |  |  |
|  | Yes | 0.93 (0.61 to 1.42, 0.732) |  |  |  |
| **Marked motor fluctuations** | No | - |  |  |  |
|  | Yes | 1.33 (0.91 to 1.96, 0.146) |  |  |  |
| **PD duration** | | 1.02 (0.98 to 1.05, 0.312) |  |  |  |
| **Admission LEDD** | | 0.98 (0.93 to 1.03, 0.398) |  |  |  |
| **Hoehn and Yahr** | 1-2 | 0.78 (0.44 to 1.40, 0.408) |  |  |  |
|  | 2.5-3 | 0.8 (0.55 to 1.17, 0.258) |  |  |  |
|  | 4-5 | - |  |  |  |
| **IMD decile** | 1 | - |  |  |  |
|  | 2 | 1.19 (0.53 to 2.69, 0.666) |  |  |  |
|  | 3 | 1.15 (0.53 to 2.53, 0.719) |  |  |  |
|  | 4 | 1.26 (0.57 to 2.80, 0.562) |  |  |  |
|  | 5 | 0.61 (0.29 to 1.31, 0.208) |  |  |  |
|  | 6 | 1.22 (0.58 to 2.61, 0.598) |  |  |  |
|  | 7 | 1.09 (0.51 to 2.34, 0.827) |  |  |  |
|  | 8 | 1.22 (0.56 to 2.64, 0.618) |  |  |  |
|  | 9 | 0.88 (0.41 to 1.88, 0.750) |  |  |  |
|  | 10 | 0.61 (0.28 to 1.32, 0.212) |  |  |  |
| **Asthma** | No | - |  |  |  |
|  | Yes | 0.87 (0.49 to 1.53, 0.624) |  |  |  |
| **Chronic pulmonary disease** | No | - |  |  |  |
|  | Yes | 1.12 (0.67 to 1.90, 0.665) |  |  |  |
| **Diabetes** | No | - |  |  |  |
|  | Yes | 1.22 (0.80 to 1.86, 0.355) |  |  |  |
| **Dementia** | No | - |  |  |  |
|  | Yes | 1.15 (0.81 to 1.62, 0.437) |  |  |  |
| **Hypertension** | No | - |  |  |  |
|  | Yes | 1.06 (0.76 to 1.49, 0.718) |  |  |  |
| **Chronic cardiac disease** | No | - |  |  |  |
|  | Yes | 1.25 (0.88 to 1.79, 0.216) |  |  |  |
| **Chronic kidney disease** | No | - |  |  |  |
|  | Yes | 1.00 (0.67 to 1.52, 0.982) |  |  |  |
| **Obesity** | No | - |  |  |  |
|  | Yes | 1.02 (0.48 to 2.16, 0.965) |  |  |  |
| **Malignant neoplasm** | No | - |  |  |  |
|  | Yes | 0.61 (0.34 to 1.10, 0.104) |  |  |  |
| **Chronic haematological disease** | No | - |  |  |  |
|  | Yes | 1.73 (0.80 to 3.94, 0.172) |  |  |  |
| **Rheumatological disorder** | No | - |  |  |  |
|  | Yes | 1.32 (0.85 to 2.06, 0.224) |  |  |  |
| **Malnutrition** | No | - |  |  |  |
|  | Yes | 1.52 (0.69 to 3.51, 0.305) |  |  |  |
| **Delirium** | No | - |  |  |  |
|  | Yes | 1.21 (0.86 to 1.72, 0.280) |  |  |  |
| **History of smoking** | No | - |  |  |  |
|  | Yes | 1.05 (0.63 to 1.74, 0.858) |  |  |  |
| **Liver disease** | No | - |  |  |  |
|  | Yes | 2.01 (0.65 to 7.50, 0.249) |  |  |  |

Abbreviations: Levodopa equivalent daily dose (LEDD), Index of multiple deprivation (IMD).
