# Supplementary material for: Clinical features and outcomes of hospitalised patients with COVID-19 and Parkinsonian disorders: A multicentre UK-based study
Source: PLoS One. 2023 Jul 31;18(7):e0285349. doi: 10.1371/journal.pone.0285349 (PMC10389727; doi:10.1371/journal.pone.0285349)
Supplement: S5 Table — (DOCX) [file pone.0285349.s007.docx]

**S5 Table:** **Univariable, multivariable and sensitivity analyses from mixed effects logistic regression models of increase in care.**

|  | | **Odds ratio**  **(95% CI, p-value)**  **Univariable** | **Odds ratio**  **(95% CI, p-value)**  **Multivariable** | **Odds ratio**  **(95% CI, p-value)**  **Multivariable, comprehensive sensitivity analysis** | **Odds ratio**  **(95% CI, p-value)**  **Multivariable, wave 2 sensitivity analysis** |
| --- | --- | --- | --- | --- | --- |
| **Acquired COVID-19** | Community | - | - | - | - |
|  | Hospital | 1.76 (1.05 to 2.96, 0.031) | 2.01 (1.10 to 3.65, 0.022) | 2.58 (1.29 to 5.15, 0.007) | 2.05 (0.95 to 4.46, 0.069) |
| **Age at admission** | | 1.01 (0.98 to 1.04, 0.395) | 1.01 (0.97 to 1.04, 0.725) | 1.01 (0.97 to 1.05, 0.593) | 1.01 (0.96 to 1.06, 0.644) |
| **Diagnosis** | Parkinson’s disease | - | - |  | - |
|  | Parkinson’s dementia syndrome | 1.09 (0.6 to 1.98, 0.773) | 0.84 (0.42 to 1.66, 0.612) | 0.78 (0.34 to 1.83, 0.574) | 0.64 (0.27 to 1.55, 0.326) |
|  | Atypical parkinsonian syndrome | 0.78 (0.26 to 2.19, 0.645) | 0.99 (0.30 to 3.26, 0.990) | 0.66 (0.17 to 2.66, 0.562) | 2.1 (0.49 to 8.92, 0.317) |
| **Ethnicity** | White British | - | - | - | - |
|  | Other | 0.6 (0.29 to 1.21, 0.166) | 0.70 (0.30 to 1.63, 0.409) | 0.80 (0.31 to 2.09, 0.655) | 0.65 (0.23 to 1.88, 0.427) |
| **Sex** | Male | - | - | - | - |
|  | Female | 1.11 (0.66 to 1.84, 0.699) | 1.10 (0.62 to 1.95, 0.745) | 0.84 (0.43 to 1.64, 0.615) | 0.80 (0.38 to 1.69, 0.554) |
| **COVID-19 wave** | 1 | 1.41 (0.8 to 2.47, 0.232) | 1.61 (0.84 to 3.09, 0.154) | - | 1.73 (0.75 to 4.02, 0.199) |
|  | 2 | - | - | - | - |
|  | Other | 0.7 (0.03 to 7.47, 0.776) | 0.22 (0.01 to 3.67, 0.290) | - | - |
| **Delirium** | No | - | - | - | - |
|  | Yes | 1.95 (1.17 to 3.29, 0.011) | 2.07 (1.48 to 3.86, 0.006) | 1.94 (1.37 to 3.99, 0.068) | 2.76 (1.67 to 7.42, 0.016) |
| **Chronic neurological disorder** | No | - |  |  |  |
|  | Yes | 0.44 (0.19 to 0.97, 0.05) |  |  |  |
| **Clinical frailty score** | <5 | 0.57 (0.26 to 1.19, 0.137) |  |  |  |
|  | 5-6 | 1.21 (0.65 to 2.26, 0.546) |  |  |  |
|  | 7-9 | - |  |  |  |
| **Hoehn and Yahr** | 1-2 | 0.27 (0.11 to 0.61, 0.002) |  |  |  |
|  | 2.5-3 | 0.6 (0.35 to 1.03, 0.065) |  |  |  |
|  | 4-5 | - |  |  |  |
| **Vaccinated** | No | - |  |  |  |
|  | Yes | 1.28 (0.39 to 4.23, 0.675) |  |  |  |
| **Significant cognitive impairment** | No | - |  |  |  |
|  | Yes | 1.1 (0.66 to 1.85, 0.707) |  |  |  |
| **Bulbar symptoms** | No | - |  |  |  |
|  | Yes | 1.06 (0.52 to 2.14, 0.865) |  |  |  |
| **Significant respiratory compromise** | No | - |  |  |  |
|  | Yes | 1.28 (0.15 to 10.78, 0.808) |  |  |  |
| **Significant autonomic neuropathy** | No | - |  |  |  |
|  | Yes | 1.26 (0.69 to 2.3, 0.451) |  |  |  |
| **Marked motor fluctuations** | No | - |  |  |  |
|  | Yes | 1.58 (0.88 to 2.86, 0.129) |  |  |  |
| **PD duration** | | 0.99 (0.95 to 1.04, 0.755) |  |  |  |
| **Admission LEDD** | | 1.02 (0.96 to 1.09, 0.468) |  |  |  |
| **IMD decile** | 1 | - |  |  |  |
|  | 2 | 0.42 (0.11 to 1.46, 0.179) |  |  |  |
|  | 3 | 1.74 (0.57 to 5.51, 0.333) |  |  |  |
|  | 4 | 2 (0.62 to 6.68, 0.249) |  |  |  |
|  | 5 | 0.53 (0.16 to 1.75, 0.301) |  |  |  |
|  | 6 | 0.98 (0.32 to 3.01, 0.975) |  |  |  |
|  | 7 | 0.89 (0.27 to 2.91, 0.845) |  |  |  |
|  | 8 | 0.95 (0.29 to 3.15, 0.936) |  |  |  |
|  | 9 | 0.98 (0.3 to 3.17, 0.97) |  |  |  |
|  | 10 | 1.67 (0.53 to 5.39, 0.384) |  |  |  |
| **Asthma** | No | - |  |  |  |
|  | Yes | 0.61 (0.24 to 1.44, 0.272) |  |  |  |
| **Chronic pulmonary disease** | No | - |  |  |  |
|  | Yes | 0.76 (0.33 to 1.67, 0.503) |  |  |  |
| **Diabetes** | No | - |  |  |  |
|  | Yes | 0.9 (0.5 to 1.6, 0.714) |  |  |  |
| **Dementia** | No | - |  |  |  |
|  | Yes | 1.44 (0.84 to 2.48, 0.188) |  |  |  |
| **Hypertension** | No | - |  |  |  |
|  | Yes | 0.94 (0.57 to 1.54, 0.805) |  |  |  |
| **Chronic cardiac disease** | No | - |  |  |  |
|  | Yes | 0.73 (0.42 to 1.24, 0.245) |  |  |  |
| **Chronic kidney disease** | No | - |  |  |  |
|  | Yes | 1.03 (0.55 to 1.91, 0.932) |  |  |  |
| **Obesity** | No | - |  |  |  |
|  | Yes | 0.83 (0.31 to 2.07, 0.686) |  |  |  |
| **Malignant neoplasm** | No | - |  |  |  |
|  | Yes | 1.13 (0.49 to 2.55, 0.77) |  |  |  |
| **Chronic haematological disease** | No | - |  |  |  |
|  | Yes | 0.56 (0.17 to 1.6, 0.303) |  |  |  |
| **Rheumatological disorder** | No | - |  |  |  |
|  | Yes | 1.36 (0.71 to 2.58, 0.351) |  |  |  |
| **Malnutrition** | No | - |  |  |  |
|  | Yes | 0.15 (0.01 to 0.86, 0.08) |  |  |  |
| **History of smoking** | No | - |  |  |  |
|  | Yes | 0.64 (0.3 to 1.35, 0.252) |  |  |  |
| **Liver disease** | No | - |  |  |  |
|  | Yes | 5.32 (0.77 to 104.88, 0.137) |  |  |  |

Abbreviations: Levodopa equivalent daily dose (LEDD), Index of multiple deprivation (IMD).
